# Incidental Memory Encoding Assessed with Signal Detection Theory and Functional Magnetic Resonance Imaging (fMRI)

**DOI:** 10.3389/fnbeh.2015.00305

**Published:** 2015-11-18

**Authors:** Benjamin Clemens, Christina Regenbogen, Kathrin Koch, Volker Backes, Nina Romanczuk-Seiferth, Katharina Pauly, N. Jon Shah, Frank Schneider, Ute Habel, Thilo Kellermann

**Affiliations:** ^1^Department of Psychiatry, Psychotherapy and Psychosomatics, Medical Faculty, RWTH AachenAachen, Germany; ^2^Department of Clinical Neuroscience, Karolinska InstitutetStockholm, Sweden; ^3^Department of Neuroradiology, Klinikum rechts der Isar, Technical University MunichMunich, Germany; ^4^TUM-Neuroimaging Center (TUM-NIC), Klinikum rechts der Isar, Technical University MunichMunich, Germany; ^5^JARA - Translational Brain MedicineAachen, Germany; ^6^Department of Psychiatry and Psychotherapy, Charité-University Medicine BerlinBerlin, Germany; ^7^Department of Neurology, Medical Faculty, RWTH AachenAachen, Germany; ^8^Institute of Neuroscience and Medicine 4, (INM 4), Forschungszentrum JülichJülich, Germany

**Keywords:** encoding, facial recognition, fMRI, memory, signal detection theory

## Abstract

In functional magnetic resonance imaging (fMRI) studies that apply a “subsequent memory” approach, successful encoding is indicated by increased fMRI activity during the encoding phase for hits vs. misses, in areas underlying memory encoding such as the hippocampal formation. Signal-detection theory (SDT) can be used to analyze memory-related fMRI activity as a function of the participant’s memory trace strength (d^′^). The goal of the present study was to use SDT to examine the relationship between fMRI activity during incidental encoding and participants’ recognition performance. To implement a new approach, post-experimental group assignment into High- or Low Performers (HP or LP) was based on 29 healthy participants’ recognition performance, assessed with SDT. The analyses focused on the interaction between the factors group (HP vs. LP) and recognition performance (hits vs. misses). A whole-brain analysis revealed increased activation for HP vs. LP during incidental encoding for remembered vs. forgotten items (hits > misses) in the insula/temporo-parietal junction (TPJ) and the fusiform gyrus (FFG). Parameter estimates in these regions exhibited a significant positive correlation with d^′^. As these brain regions are highly relevant for salience detection (insula), stimulus-driven attention (TPJ), and content-specific processing of mnemonic stimuli (FFG), we suggest that HPs’ elevated memory performance was associated with enhanced attentional and content-specific sensory processing during the encoding phase. We provide first correlative evidence that encoding-related activity in content-specific sensory areas and content-independent attention and salience detection areas influences memory performance in a task with incidental encoding of facial stimuli. Based on our findings, we discuss whether the aforementioned group differences in brain activity during incidental encoding might constitute the basis of general differences in memory performance between HP and LP.

## Introduction

Although human beings encounter a plethora of everyday events, subjective experiences, and affective states, not all of these are transformed into permanent memories. Throughout the last two decades, cognitive neuroscientists have employed various methods, ranging from animal behaviorism to functional imaging studies, to disentangle how we form and retrieve permanent memories, which essentially make up the core of our personal history and individual identity. In order to compare events that will later be remembered to those that will be forgotten, a widely used experimental design was developed in the context of event-related functional magnetic resonance imaging (fMRI). It employs an encoding phase, during which stimuli such as words or pictures are presented, and a recognition phase, or subsequent memory test, during which participants have to indicate which stimuli have already been presented in the encoding phase and which ones are new. Typically, a comparison and respective blood oxygenation level dependent (BOLD) activation contrast is based on later performance levels (remembered vs. forgotten; Brewer et al., 1998; Wagner et al., 1998; Kim et al., 2010). Generally, increased BOLD activation for later remembered compared to later forgotten items indicates successful encoding, whereas the reverse activity pattern indicates neural activity interfering with successful encoding (Wagner et al., 1998; Kirchhoff et al., 2000; Uncapher et al., 2006). However, decreased BOLD activity, located within medial and lateral parietal areas comprising the default mode network (DMN), has also been related to successful memory encoding (Daselaar et al., 2004; Anticevic et al., 2010). Whereas increased BOLD activations most likely relate to the actual implementation and performance of various mnemonic operations, deactivations may represent a more general downregulation of the DMN: as a prerequisite for successful memory encoding, the areas comprising the DMN must be deactivated, in order to enable sustained cognitive effort, task performance and the formation of mnemonic representations (Miller et al., 2008; Anticevic et al., 2010).

The goal in many previous studies was to compare encoding-related activation to recognition-related activation, as well as to explore a recognition effect (i.e., contrasting old vs. new mnemonic material). A network associated with encoding and recognition during subsequent memory tasks includes the prefrontal cortex (PFC), middle temporal lobe (MTL; Buckner et al., 2001; Fernández and Tendolkar, 2001; Simons and Spiers, 2003), fusiform gyrus (FFG; Dickerson et al., 2007; Kim and Cabeza, 2007), posterior parietal cortex (PPC; Sommer et al., 2005; Uncapher and Rugg, 2009), and the premotor cortex (PMC; Morcom et al., 2003; Kao et al., 2005). These brain regions can be clustered into three types of activation foci (de Chastelaine and Rugg, 2014; Kim, 2011): content-specific regions including the inferior frontal cortex (IFC) and the FFG are primarily responsible for encoding and transforming sensory input into internal representations (Paller and Wagner, 2002). Storage regions, such as the MTL and the hippocampal formation, subsequently bind these internal representations into a constant memory which is accessible for later conscious retrieval (Squire et al., 2004; Diana et al., 2007). Finally, attentional processing during encoding, sub-served by the PMC and parietal regions such as the PPC, might be employed to bias towards a specific event so that it can be selected among competing input (Kim, 2011). Regarding the specific impact of emotional material on memory, it is well known that emotionally arousing stimuli enhance memory performance (Bradley et al., 1992; Chiu et al., 2013), which, on a neural level, is associated with increased activity of the amygdala and the hippocampal formation during both encoding and retrieval (Dolcos et al., 2004, 2005, 2012; Kensinger and Schacter, 2005; Murty et al., 2010; Shafer and Dolcos, 2014).

**Figure 1:**
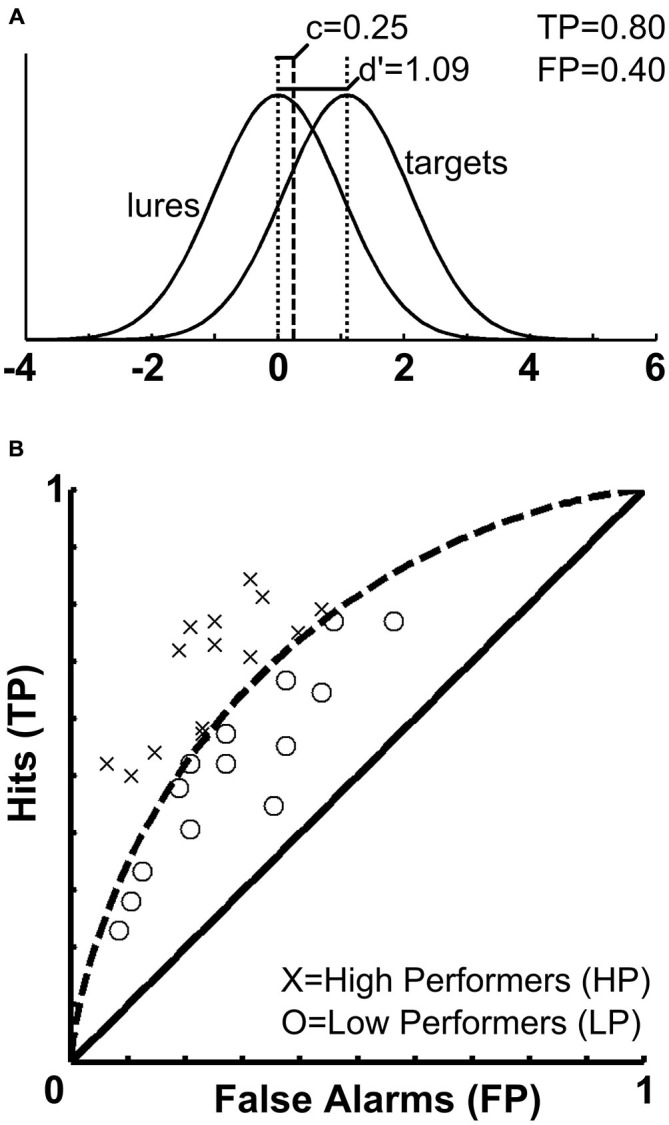
**Signal strength, decision criterion and receiver operating characteristics. (A)** The upper panel illustrates the distribution of memory trace strength (d^′^) and decision criterion (c) in the current sample. Whereas (d^′^) is used to divide participants according to their behavioral performance and assess correlations with brain activity, (c) is used a covariate in the fMRI analyses to account for variance related to potential response bias. **(B)** The lower panel depicts the receiver operating characteristics (ROCs) space for the current sample. Based on the memory trace strength (d^′^), it was established that participants were categorized as HPs if they correctly classified at least 54 of the 96 old faces and at the same time, correctly rejected at least 27 of the 48 new faces. Accordingly, the curved dashed line separates the sample into HP and LP.

As an alternative to a classical investigation of memory performance involving solely the comparison of remembered vs. forgotten stimuli, signal-detection-theory (SDT) can be employed in order to separate participants based on a certain performance outcome measure. The theoretical considerations and behavioral measures resulting from SDT are employed in psychological research in order to determine how humans make decisions under conditions of uncertainty, for example, when asked to decide whether a certain stimulus has been previously presented or not. Studies applying SDT to examine memory processes have revealed that “[…] recognition decisions are based on the strength of a memory signal in relation to a decision criterion” (Wixted, 2007). According to Yonelinas and Parks (2007), these SDT approaches developed with studies using receiver operating characteristic (ROC) analyses. These relate the proportion of correctly recognized old items (i.e., hits) and incorrectly recognized new lure items (i.e., false alarms). The signal or memory strength (d^′^) refers to the distance between the distributions of lures and targets, and the underlying distributions are assumed to be of Gaussian nature and—in the simplest case—of equal variance. ROC functions also take into account a decision criterion (c) which denotes a memory signal strength threshold. If a specific test item generates memory strength exceeding the criterion it is declared to be old; otherwise it is declared a new item (Wixted, 2007). For each individual participating in a memory experiment, both quantities (i.e., signal strength and decision criterion) can be calculated from a 2 × 2 contingency table consisting of true positives (TP, or hits), true negatives (TN), false positives (FP, or false alarms) and false negatives (FN, or misses). Presenting data from the current sample, the aforementioned measures of SDT are further illustrated in Figure 1. Brain-imaging studies taking into account participants’ behavioral performance levels and ROC analyses are based on the assumption that memory-related brain activation varies as a function of the memory trace strength (d^′^). In previous studies, brain activations during encoding and recognition phases were each analyzed as a function of the participant’s behavioral performance during the recognition phase, and the resulting activations and deactivations were subsequently related to whether they were beneficial or detrimental to memory performance (Paller and Wagner, 2002; Daselaar et al., 2004; Uncapher and Wagner, 2009). However, such an approach so far has not been tested for incidental encoding of facial stimuli.

Thus, we followed and extended the subsequent memory approach and analyzed fMRI activity during incidental encoding of facial stimuli in relation to the participants’ subsequent behavioral recognition performance determined by SDT. Using an incidental encoding memory task seemed an appropriate choice for several reasons. First, to our knowledge, there have not been any attempts to examine correlations between brain activity obtained during incidental encoding of facial stimuli and subsequent behavioral recognition performance. More importantly, the incidental encoding task is assumed to possess high ecological validity as it resembles the formation of memories during everyday life, where instructions to remember certain stimuli are rarely present. While brain activity patterns associated within incidental and intentional encoding tasks largely overlap (Rugg et al., 1997), including areas relevant for memory encoding and recognition such as MTL, prefrontal, parietal, premotor and sensory regions, the right PFC was reported to be specifically active for intentional encoding, most likely due to its role in controlling retrieval effort and verification of retrieved information.

Our aim was to demonstrate that recognition performance of facial stimuli would be significantly correlated to incidental encoding-related brain activity. In order to avoid several regression approaches involving multiple independent variables (Vul et al., 2009) and to provide a new approach in fMRI memory research incorporating ROC analyses we split our sample into two groups based on memory strength (d^′^) and contrasted brain activity for high performers (HP) with that of low performers (LP). Thus, our study examines for the first time whether a post-experimental group assignment based on SDT measures of recognition performance interacts with fMRI activity during incidental encoding of facial stimuli. Our hypotheses were specifically related to this post-experimental group assignment and the interaction with fMRI activation patterns. We hypothesized that HP would show increased activity in brain areas associated with successful face encoding compared to LP. Furthermore, we expected the relationship between behavioral performance and brain activity to be present in both content-specific regions and brain areas responsible for attentional processing during encoding. This hypothesis was based on the assumption that both increased attentional and content-specific processing would be beneficial for subsequent memory performance and might constitute a key difference between HP and LP.

## Materials and Methods

### Participants

Twenty-nine healthy volunteers (mean age 34.31 years, *SD* = 9.29; 15 females) were recruited by means of local advertisements followed by a detailed screening. All participants were right-handed (Oldfield, 1971), had normal or corrected-to-normal vision, no contraindications against MR measurements, and no history of neurological or psychiatric illness or any other disorder which might affect cerebral metabolism. Participants were excluded in case of current substance abuse, based on a urinal drug screening. Furthermore, all participants had the same educational level (German A-levels). All experimental procedures were approved by the Ethics Committee of the Medical Faculty of the RWTH Aachen University and performed in compliance with the Code of Ethics of the World Medical Association (Declaration of Helsinki). All participants gave their written informed consent and received compensatory payment.

### Experimental Task and Procedures

In order to investigate a subsequent memory effect with facial stimuli we used a modified version of the Facial Emotions for Brain Activation (FEBA) test (Gur et al., 2002; Schneider et al., 2006), which consisted of colored photographs depicting 96 male and female faces (48 each) with different facial expressions (happy, sad, angry, fearful, or neutral), balanced with regard to age, sex and ethnicity. This stimulus set has already been applied in several previous studies (Gur et al., 2002; Schneider et al., 2006; Seiferth et al., 2008, 2009; Habel et al., 2010).

**Figure 2:**
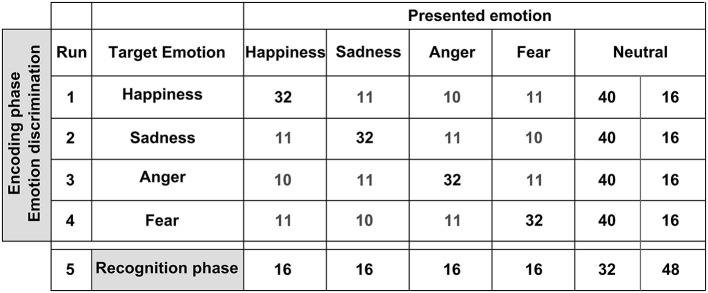
**Stimuli employed for the subsequent memory task.** We used a modified version of the Facial Emotions for Brain Activation (FEBA) test. The upper panel depicts the encoding phase during which stimuli were presented in four runs. Within each run, one of the four emotions was the target emotion and 120 FEBA pictures were presented. The participants’ task during the incidental encoding phase was to indicate with their left index finger if the stimulus met the designated target emotion and to indicate with their right index finger if the stimulus displayed any other emotional expression or neutral. The lower panel depicts the recognition phase. Ninety six neutral pictures of the formerly emotionally presented target faces (4 × 16) and the formerly neutrally presented faces (32) were presented, as well as 48 pictures of new neutral faces. The participants’ task was to indicate with their left index finger if a facial stimulus had been presented before and with their right index finger if the facial stimulus showed a new person.

The event-related fMRI experiment consisted of an incidental encoding phase (masked as an emotion discrimination task), and a recognition phase, as depicted in Figure 2. During encoding, stimuli were presented in four runs (order randomly assigned). In each run, one of the four emotions was the target emotion and 120 FEBA pictures were presented: 32 faces showed the target emotion, 32 showed the non-target emotions, and 56 faces showed a neutral expression (see Figure 2). These neutral faces included persons shown before with emotional facial expressions, as well as new neutral faces. Participants therefore saw each face five times across the four encoding runs. The participants’ task during the incidental encoding phase was to indicate with their left index finger if the stimulus met the designated target emotion and to indicate with their right index finger if the stimulus displayed any other emotional or neutral expression. This was followed by a recognition task during which all actors which had been shown in runs 1–4 were presented again. Here, 96 neutral pictures of the formerly emotionally presented target faces (4 × 16) and the formerly neutrally presented faces (32) were presented, plus 48 pictures of new neutral faces, so-called “lures” (totaling to 144). The participants’ task during the recognition phase was to indicate with their left index finger if a facial stimulus had been presented before and with their right index finger if the facial stimulus showed a new person. All stimuli were presented for two seconds (ISI 1 s, fixation cross; null-events 1.5–4.5 s, jittered) using Presentation 0.70 software (Neurobehavioral Systems Inc., San Francisco, CA, USA). The response buttons were located on an MR compatible response system (LUMItouch, Lightwave Technologies, Richmond, Canada). For further and more detailed descriptions of the task please refer to one of our previous publications (e.g., Habel et al., 2010).

Moreover, participants were tested with a neuropsychological battery, including tests on attention, working memory, visuomotor information processing and executive functioning (Continuous Performance Test, CPT; Weintraub and Mesulam, 1985; Trail Making Test, TMT; Reitan, 1992), face processing (Benton Facial Recognition Test, BFRT; Benton et al., 1994), facial emotion recognition (Penn Emotion Recognition Test, PERT 40; Kohler et al., 2004), and global functioning level (Global Assessment of Functioning Scale, GAF, in Structured Clinical Interview for DSM-IV; Wittchen et al., 1997). Regarding neuropsychological data, HP and LP did not differ significantly (all *p* > 0.05). Table 1 displays demographic and neuropsychological data of all participants.

**Table 1:** **Demographic and neuropsychological characteristics of the sample**.

	All (*N* = 29)	HP (*N* = 15)	LP (*N* = 14)	*T*	*p*
Age (years)	34.31 ± 9.29	34.27 ± 9.52	34.36 ± 9.35	−0.026	0.980
Education (years)	12.79 ± 3.06	12.86 ± 3.26	12.71 ± 2.87	0.121	0.904
TMT	24.50 ± 6.49	23.36 ± 5.89	25.64 ± 7.09	−0.930	0.361
CPT (RT verbal)	526.36 ± 77.82	524.93 ± 58.84	527.79 ± 97.91	−0.095	0.925
CPT (RT spatial)	494.54 ± 51.90	501.07 ± 41.00	488 ± 63.58	0.658	0.516
BFRT	42.07 ± 15.16	44.29 ± 12.88	39.86 ± 17.41	0.767	0.450
PERT (hits)	31.18 ± 6.59	31.64 ± 2.61	30.71 ± 9.44	0.367	0.717
PERT (RT)	2405.68 ± 866.00	2512.21 ± 896.33	2299.14 ± 841.77	0.644	0.525
GAF	86.43 ± 17.04	82.86 ± 23.20	90.00 ± 0.00	−1.114	0.276

*BFRT, Benton Facial Recognition Test; CPT, Continuous Performance Test; GAF, Global Assessment of Functioning Scale; HP, high performers; LP, low performers; PERT, Penn Emotion Recognition Test; TMT, Trail Making Test*.

### Analyses of Behavioral Performance

We aimed at avoiding several regression approaches involving multiple independent variables (d^′^ and the c criterion) and multiple non-independent BOLD contrast images (hits > misses, hits > baseline, misses > baseline) for two reasons. On the one hand, non-independent BOLD contrasts involve the problem of circularity (Vul et al., 2009) and increase the number of tests performed. On the other hand, such independently performed whole-brain regression analyses may lead to inconsistent results. We decided to identify regions of interest based on an interaction between behavioral memory performance and BOLD responses for remembered (hits) and forgotten (misses) items. Therefore, the sample was split into two groups based on individual recognition performance. Participants were assigned to the high-performers (HP, *n* = 15) or low-performers group (LP, *n* = 14), respectively. The cut-off value to be assigned to the HP group was fixed at a minimum of 81 correct responses out of 144 possible (56.25%). According to the binomial distribution, the probability of such an event to occur at random is *p* < 0.0782, assuming a chance of a correct answer of 50% for each presented face. In order to prevent effects from any kind of response bias we chose to apply the threshold of 56.25% simultaneously to old and new faces. Accordingly, participants were categorized as HPs if they correctly classified at least 54 of the 96 old faces and at the same time correctly rejected at least 27 of the 48 new faces. For a graphic illustration of the ROC curve and the SDT measures obtained from the current sample, please refer to Figure 1.

### Image Acquisition

Functional MRI measurements were performed on a Siemens 1.5 Tesla Sonata MRI scanner (Siemens AG; Erlangen, Germany) using an 8-channel head matrix coil. To stabilize the position of the head during scanning, foam pads were used. Each participant underwent five functional runs. One hundred forty-three functional images were acquired for each of the four encoding runs, whereas 167 functional images were acquired for the recognition run. For all runs, a single shot pulse EPI sequence with the following acquisition parameters was used: *TR* = 3000 ms, flip *angle* = 90°, *FOV* = 200 × 200 mm^2^, matrix *size* = 64 × 64, 30 slices parallel to the anterior/posterior commissural plane, slice *thickness* = 3 mm, 0.3 mm gap, voxel *size* = 3.125 × 3.125 × 3 mm^3^. High-resolution anatomical images were acquired for each participant using an MPRAGE sequence with the following acquisition parameters: *TR* = 2200 ms, *TE* = 4 ms, flip *angle* = 15°, *FOV* = 256 × 256 mm^2^, 160 sagittal slices, voxel *size* = 1 × 1 × 1 mm^3^.

### Image Processing

Preprocessing of functional data and all further image analyses were done using SPM5 (Institute of Neurology, London, UK; www.fil.ion.ucl.ac.uk/spm). The first three volumes of each functional time series were discarded, preventing artifacts from transient signal changes at the beginning of each functional run until the brain reaches a stable magnetized state. Functional images were realigned to the first image using affine spatial transformations and a least-squares approach. We verified that none of the participants exceeded the predefined movement limits of 3 mm, or 3°. After slice-time correction, the mean functional image was estimated and used as the reference image for co-registration of the individual high-resolution anatomical image of each participant. Anatomical images were normalized to a standard T1-template image (5th degree spline interpolation to 2 × 2 × 2 mm^3^ resolution). These normalization parameters were then applied to the functional time series. Finally, functional images were spatially smoothed using a 10 mm FWHM Gaussian kernel to account for inter-subject variability.

Regarding statistical analyses, it should be noted that for the present study, we focused on the activity during the encoding phase, following previous subsequent memory studies (Brewer et al., 1998; Wagner et al., 1998; Kirchhoff et al., 2000; Garoff et al., 2005; Kim et al., 2010). We first defined first-level general linear models (GLM) for each participant. All four encoding runs containing the different target emotions were modeled together, because the present study explicitly focused on memory-related group differences based on behavioral performance and SDT measures (for differences regarding different target emotions see Habel et al., 2010). Delta functions of the stimulus onset times were convolved with the canonical hemodynamic response function implemented in SPM5, and subsequently entered in the first-level GLMs as regressors of interest. T-contrast images were obtained for the following contrasts: hits vs. (implicit) baseline, and misses vs. (implicit) baseline, and included in a second-level 2 × 2 ANOVA with the between-subject factor “group” (HP vs. LP) and the within-subject factor “recognition” (hits vs. misses). The decision criterion (c) was included as a covariate in the model to account for variance related to response bias. As our goal was to specifically examine group differences in the hits > misses contrast, we focused the analysis on the interaction between “group” and “recognition” using the t-contrasts HP vs. LP and *vice versa*. To correct for multiple comparisons, an uncorrected voxel-level threshold of *p* = 0.001 (*t* = 3.2) was set, and the thresholded maps were subsequently submitted to a whole-brain correction based on an iterative procedure (Monte Carlo simulation) used to estimate cluster-level false-positive rates and the spatial smoothness of the functional data using AlphaSim (Ward, 2000). After 10,000 iterations, the minimal cluster-size threshold yielding a cluster-level false-positive rate of 5% was determined to be *k* = 125 voxels. The cluster-size threshold (*k* = 125 voxels) was applied to the statistical maps, and in combination with the voxel-level threshold (*p* = 0.001) resulted in an estimated whole-brain corrected α = 5% level. The relationship between behavioral performance and brain activity was tested in all areas resulting from the aforementioned contrasts, by correlating d^′^ with parameter estimates of hits vs. misses, hits vs. baseline, and misses vs. baseline across the entire sample using Pearson’s correlation coefficient, with Bonferroni-corrected *p*-values.

## Results

### fMRI Results

**Figure 3:**
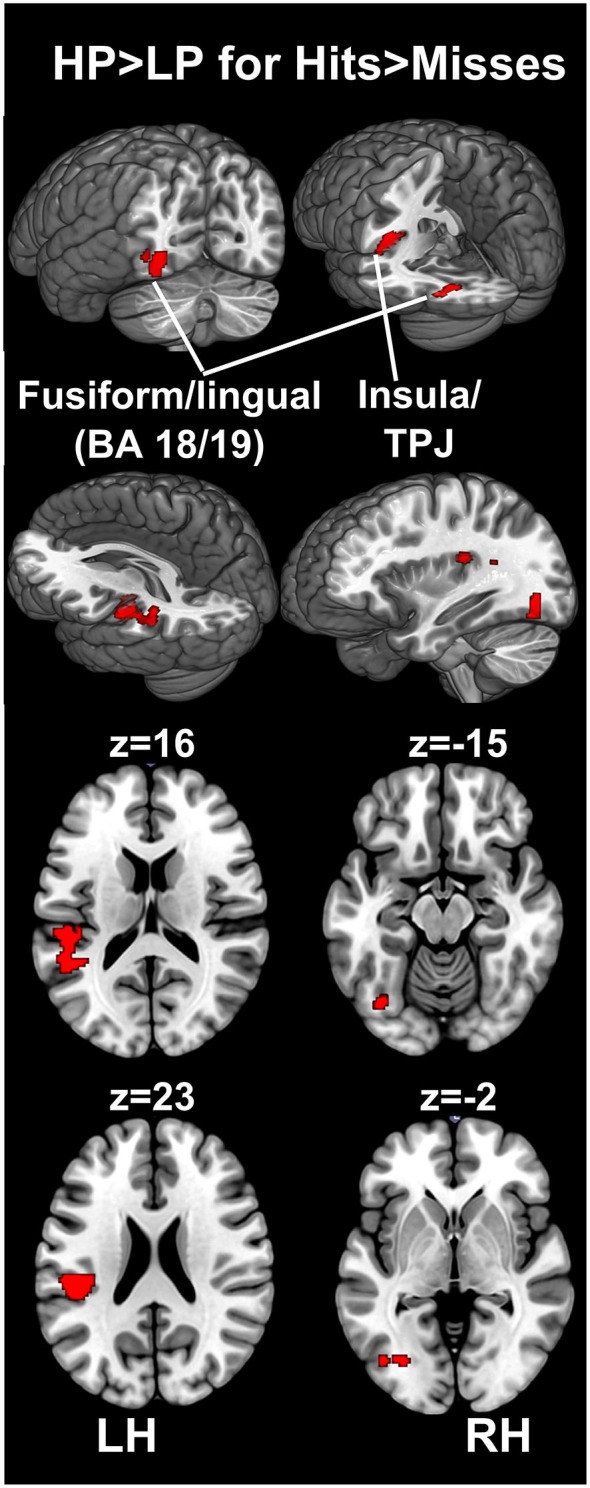
**fMRI results.** The figure illustrates differences between the HP and the LP group for the contrast hits > misses. To correct for multiple comparisons, an uncorrected voxel-level threshold of *p* = 0.001 (*t* = 3.2) was set, and in combination with a cluster-size threshold of *k* = 125 voxels resulted in an estimated whole-brain corrected α=5% level. The cluster-size threshold was determined using Monte Carlo simulations (10,000 iterations). Increased activity for the HP group was found in the left FFG, covering both BA 18 and 19, including parts of the fusiform and the lingual gyrus. Furthermore, increased activity for the HP group was found in the left insular cortex. This cluster covered mainly the posterior insula (BA 13) and extended towards the ventral part of the TPJ at BA 40. *BA, Brodmann area; FFG, fusiform gyrus; HP, high performers; LH, left hemisphere; LP, low performers; RH, right hemisphere; TPJ, temporo-parietal junction*.

**Figure 4:**
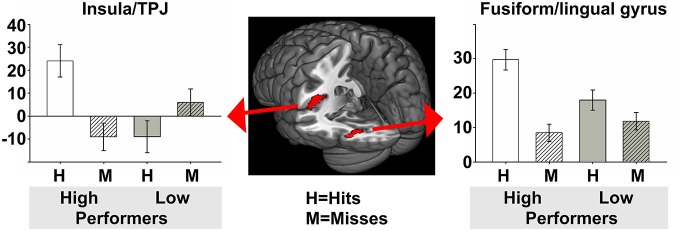
**Parameter estimates for the two activated clusters.** To further illustrate group differences between HP and LP, this figure depicts the parameter estimates including 90% confidence intervals, for hits and misses, for both the HP and the LP group. In both clusters resulting from the contrast HP > LP for hits > misses the HP group had more activity for hits than for misses, and more activity for hits than the LP group. *HP, high performers; LP, low performers*.

For the encoding of mnemonic material later remembered, our analysis resulted in activations present in two cortical regions, namely the insula/temporo-parietal junction (TPJ) and the FFG of the left hemisphere. This analysis revealed an interaction between post-experimental group assignment and recognition performance, or in other words increased activation for the HP group compared to the LP group, for the contrast hits > misses in two activation clusters. The insula/TPJ cluster covered both Brodmann Area (BA) 13 and BA 40, and was located at the posterior part of the insula, covering also ventral aspects of the TPJ. The FFG cluster comprised both BA 18 and BA 19 and covered mainly the more dorsally located FFG, but also small parts of the more ventrally located lingual gyrus. A detailed summary of the activated clusters, including peak coordinates, T values, cluster sizes, and effects sizes (Cohen’s d; Cohen, 1988) can be found in Table 2 and the results are visualized in Figure 3. As can be seen from the parameter estimates presented in Figure 4, HP showed higher activity both in the FFG and in the insula/TPJ for hits as compared to misses. Importantly, HP also showed more activity for hits than the LP participants in both clusters. The reverse contrast, LP > HP for hits > misses did not reveal significant activation.

**Table 2:** **Overview of fMRI results**.

Region	BA	*X*	*Y*	*Z*	*T*-Value	No. of voxels	Effect size (Cohen’s *d*)
insula/TPJ	13/40	−46	−30	20	4.96	356	1.35
fusiform/lingual gyrus	18/19	−32	−72	−6	3.7	126	1.02

*An uncorrected voxel-level threshold of p = 0.001 (t = 3.2) was set, and in combination with a cluster-size threshold of k = 125 voxels resulted in an estimated whole-brain corrected α = 5% level. The cluster-size threshold was determined using Monte Carlo simulations (10,000 iterations). The X, Y, and Z coordinates refer to the MNI coordinate system. The standard formula for Cohen’s d (d = 2t/√(df)) was used to calculate effect sizes. BA, Brodmann area; TPJ, temporo-parietal junction*.

### Correlation between Recognition Performance and fMRI Activity During the Encoding Phase

In order to investigate brain-behavior associations we correlated extracted parameter estimates from three different contrasts with d^′^ for each participant. Parameter estimates were extracted for the contrasts hits > misses, hits > baseline, as well as misses > baseline from the peak voxel of the two clusters activated in the HP > LP for hits > misses contrast (cf. Table 2). These six values per subject were then correlated with d^′^ using Pearson’s correlation coefficient. Since we compared 6 correlation coefficients in total (3 different contrasts for 2 clusters), the significance level was adjusted using Bonferroni correction, resulting in an adjusted *p* < 0.0085. Because we expected positive correlations for two contrasts (hits > misses and hits > baseline) and negative correlations for one contrast (misses > baseline) these tests were performed unidirectionally.

**Figure 5:**
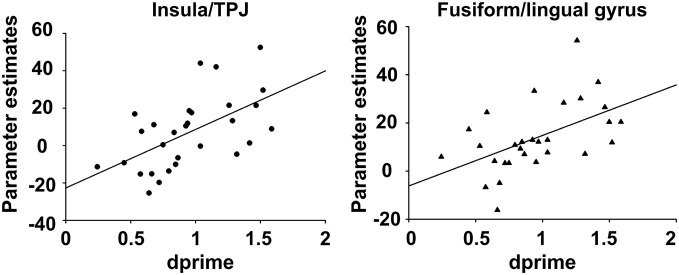
**Correlation between behavioral recognition performance and fMRI encoding activity.** The figure depicts the correlation between behavioral recognition performance, as assessed with memory trace strength (d^′^), and fMRI activity, for the contrast (hits > misses) during the encoding phase of the subsequent memory task. For both clusters, increased activity for (hits > misses) was positively correlated to better recognition performance.

The insula/TPJ and the FFG exhibited a significant positive correlation of incidental encoding-related brain activity (hits > misses) with recognition performance (d^′^; see Table 3). The correlation was comparable for the insula/TPJ cluster (*r* = 0.56; *p* = 0.0012) and the FFG cluster (*r* = 0.52; *p* = 0.0039). All remaining correlations between brain activity and behavioral performance were not significant. The results for the correlations between recognition performance and fMRI activity are summarized in Table 3 and illustrated in Figure 5.

**Table 3:** **Correlations of behavioral recognition performance as assessed with SDT and fMRI activity**.

Region	Correlation with hits > misses	Correlation with hits > baseline	Correlation with misses > baseline
insula/TPJ	*r* = 0.56 (*p* = 0.0012)*	*r* = 0.34 (*p* = 0.0725)	*r* = −0.29 (*p* = 0.1323)
fusiform/lingual gyrus	*r* = 0.52 (*p* = 0.0039)*	*r* = 0.44 (*p* = 0.0143)	*r* = 0.03 (*p* = 0.8266)

*The (r) refers to the Pearson correlation coefficient. The asterisk indicates significant correlations at a Bonferroni corrected p-value of p = 0.0085, which was used to correct for multiple comparisons. r, Pearson’s correlation coefficient; TPJ, temporo-parietal junction*.

## Discussion

Previous fMRI studies have successfully employed a subsequent memory approach to identify and localize patterns of brain activity specifically related to encoding of stimuli later remembered, as compared to those later forgotten. All such studies have indirectly incorporated behavioral performance, since encoding activity was analyzed based on participants’ performance during the recognition task. However, up to date it remained unclear whether encoding activity correlated with recognition performance also in an incidental encoding memory task. To shed light on this question, the present fMRI study combined SDT and a subsequent memory task on incidentally encoded visual material in a group of healthy volunteers. We used the standard indicator d^′^ from SDT to split our sample according to individual recognition performances, resulting in a HP and a LP group. Thus, we examined whether post-experimental group assignment based on recognition performance would relate to fMRI activity corresponding to incidental encoding of emotional and neutral faces. Because the effects of the different target emotions of the task employed here was already evaluated in previous studies (e.g., Habel et al., 2010), and because we wanted to focus explicitly on using SDT measures to examine the relationship between brain activity during incidental encoding and recognition performance, our analyses do not differentiate between the four target emotions. Thus, we were only interested in the relationship between brain activity during general, incidental encoding of visual stimuli, irrespective of their content, and subsequent recognition performance.

We demonstrated that post-experimental group assignment and therefore memory performance itself was significantly associated with fMRI activity during successful incidental encoding in the insula/TPJ and in the FFG, with an advantageous processing of remembered compared to forgotten items in the HP compared to the LP group. Our results revealed a significant interaction between group and recognition performance in the sense that the HP group exhibited increased activity in the hits > misses contrast for the insula/TPJ and in the FFG. As illustrated in Figure 4, parameter estimates clearly revealed that HP participants exhibited more activity than LP participants in both clusters for hits, indicating differential processing of remembered items in the HP group. Extending the results of previous studies (Kirchhoff et al., 2000; Paller and Wagner, 2002; Sperling et al., 2003; Dickerson et al., 2007), we were able to demonstrate that activity within these two clusters showed a significant positive correlation with behavioral recognition performance. Thus, the more activity participants showed in the hits > misses contrast the better their recognition performance was. With the present study, we thus provide evidence that, besides activity within storage regions such as the MTL and the hippocampus, encoding activity in content-specific sensory areas and content-independent attention and salience detection areas directly influences memory performance.

### Insula and TPJ Activity

With regard to activation differences in the insula and the insula’s correlation with recognition performance, we suggest that HP’s ability to remember more stimuli was directly associated with increased activity in this salience detection area: higher insula activity during the incidental encoding phase might have enabled HP to mark a higher number of stimuli as salient and thus remembered more stimuli correctly during the recognition task. From previous neuroimaging studies it is well known that the insula is a functionally heterogeneous brain area, with assigned functions ranging from visceral, sensorimotor, interoceptive, autonomic and homeostatic processing, to response selection, emotional self-awareness and motor control of speech production (Dronkers, 1996; Cereda et al., 2002; Craig, 2002; Critchley, 2005; Seminowicz and Davis, 2007; Taylor et al., 2008). Because of its unique anatomical location, this region strongly interacts with limbic, somatosensory, linguistic as well as motor regions (Augustine, 1996; Menon and Uddin, 2010; Uddin, 2015) and despite the various potential functions that the insula might sub-serve in cooperation with other brain regions, there is relatively little doubt that one of the core functions of the insular cortex *per se* is salience detection, or in other words to detect and increase attention towards salient or new stimuli, marking and highlighting them for further processing (Seeley et al., 2007; Menon and Uddin, 2010; Uddin, 2015). Previous neuroimaging studies (Peyron et al., 2000; Blood and Zatorre, 2001; Craig, 2002; Kim, 2014), containing musical, painful and metabolic stimuli, have shown the insula’s salience-sensitivity across different modalities and specifically emphasized an important role of salient facial expressions (Bartels and Zeki, 2004; Singer et al., 2004). With regard to different insula sub-divisions, several fMRI studies suggested that the present posterior insula activation may be more responsible for general environmental monitoring (i.e., salience detection) and response selection, whereas the anterior insula may be more responsible for subjective evaluations of internal conditions and emotional awareness (Craig, 2002; Taylor et al., 2008; Ebisch et al., 2011). This distinction obtained by resting-state fMRI studies is also supported by anatomical connectivity: in order to sub-serve functions such as salience detection and response selection, the posterior insula must receive direct input regarding homeostatic afferent information, which is provided by the thalamocortical pathway (Augustine, 1996; Craig, 2002; Saper, 2002). With respect to the present study, we specifically agree with the results presented by Taylor et al. (2008), who suggest that the bilateral mid-posterior insula is responsible for detection of salient perceptual stimuli and response selection. We conclude that higher posterior insula activity most likely enhanced response selection and salience detection in HP, thus contributing to their superior recognition performance. Increased posterior insula activity for hits > misses in HP may have therefore increased reactivity to the hits and thereby initiated differential processing of these stimuli, which might have subsequently resulted in better memory for these specific stimuli. This is in line with the fact that participants performed an *incidental* encoding task, because salience detection and marking salient stimuli for further processing is a task-independent function of the posterior insula. An alternative explanation for the insula activity might be its involvement in emotional learning and the evaluation of emotional outcomes (Büchel et al., 1999; Bar-On et al., 2003; Paulus et al., 2003).

The activation cluster did not only incorporate the insular cortex, but also the ventral part of the left TPJ (BA 40). It is well known from previous neuroimaging studies that the right TPJ is involved in stimulus-driven attention (Corbetta et al., 2008; Shulman et al., 2009; Clemens et al., 2011, 2013). This also holds true for the left TPJ, specifically during working memory tasks. Ravizza et al. (2011) found increased left TPJ activation during both encoding and retrieval phases, with the peak left TPJ coordinate located close to the insula/TPJ peak coordinates found in the present study. They concluded that the left TPJ might provide the necessary attentional resources during memory tasks, sub-serving a comparable function to its right homolog. In accordance with the aforementioned interpretation we suggest that HP exhibited more attention to salient stimuli, which was evident in increased activation of the left TPJ, specifically enabling HP to later remember more items correctly, which in turn resulted in better recognition performance. We provide here experimental evidence that this interpretation also holds true for incidental memory encoding of facial stimuli.

In addition to providing beneficial attentional resources, the left TPJ has also been implicated, along with the insula, in the detection of salient events in the environment (Downar et al., 2000). Previous memory studies (Hayama et al., 2012; Rugg and Vilberg, 2013) suggested that such left insula/TPJ activations represent content-independent activity, which might be engaged irrespectively of the stimulus nature and task instructions. We conclude that our findings are in agreement with previous studies, and we extend them by showing a positive correlation between activity of the left insula/TPJ, during incidental encoding of facial stimuli, and subsequent behavioral recognition performance. The observed correlations may be cautiously interpreted such that activation differences in content-independent brain areas influence memory performance. Thus, in the typically noisy environment of the incidental encoding phase, increased activation in content-independent areas might help to focus attention and refine salience detection, which in turn might help to reduce the amount of noise and isolate stimuli which can later be successfully remembered.

### FFG Activity

The left FFG is specifically involved in memory encoding of facial stimuli (Bi et al., 2014), has been shown to underlie specific aspects of face analyses (Meng et al., 2012), but is also coding specifically for emotionality, as presented in a large fMRI meta-analysis (Fusar-Poli et al., 2009). Beyond the FFG which is specifically responsive to faces (Kanwisher et al., 1997; McCarthy et al., 1997; Grill-Spector et al., 2004; Fang et al., 2007; Axelrod and Yovel, 2012; Zhang et al., 2012), the occipital face area (OFA) and the posterior superior temporal sulcus (pSTS) comprise the core face recognition network (Haxby et al., 2000; Rossion et al., 2003; Collins and Olson, 2014). Overall, the FFG contains functionally different and heterogeneous visual areas: whereas more posterior areas around area V4 are involved in early visual processing (Rottschy et al., 2007; Wilms et al., 2010), more anterior parts contain the aforementioned face-selective area (Kanwisher et al., 1997). With respect to the present results, Garoff et al. (2005) demonstrated that specifically the left FFG, as opposed to its right homolog, has a specific role in memory. They used a modified subsequent memory task to show that encoding-related activity in the left FFG resulted in more general and holistic, and thus non-specific recognition. In a recent study, Bi et al. (2014) presented fMRI and cortical thickness measures to confirm that both structure and function of the left FFG are closely associated with perceptual learning of faces. Further evidence for an involvement of the left FFG in creating memories of faces is obtained from two event-related potential (ERP) studies, showing left-lateralized ERP changes, which were localized to occipital-temporal areas for perceptual learning of facial stimuli (Rossion et al., 2002; Su et al., 2012). Taken together, the aforementioned findings indicate that learning and differences in mnemonic abilities can influence content-specific left FFG activity and *vice versa*. This fits with our interpretation of the present results, namely that the differences in left FFG activity during incidental encoding directly relate to behavioral differences in memory performance, as indicated by the correlations between encoding activity in the left FFG and recognition performance. Importantly, we provide correlative evidence that such a relationship also exists if an incidental encoding task is employed.

Furthermore, activity within the FFG during encoding phases of memory tasks corroborates previous results from subsequent memory fMRI studies (Dickerson et al., 2007; Kim and Cabeza, 2007), which have conceptualized this area as content-specific. Previous fMRI studies suggested that FFG activity during intentional encoding predicts subsequent recognition performance (Brewer et al., 1998; Wagner et al., 1998; Kirchhoff et al., 2000; Paller and Wagner, 2002; Sperling et al., 2003; Dickerson et al., 2007). We extend these findings by using post-experimental group assignment based on SDT to show that FFG activity during *incidental* encoding specifically correlates with the difference between remembered vs. forgotten items. We suggest that the main task of the FFG during incidental memory encoding might have been to process sensory and perceptual features of faces, and transform this basic input into internal representations which can subsequently be used for further operations such as storage and recognition. Increased FFG activity during incidental encoding might thus help HP to create more detailed and precise internal representations of the stimuli to be encoded, which in turn might provide prerequisites for better performance of HP during recognition. More specifically, the FFG’s involvement might have enabled participants to recapitulate prototypical and crucial visual properties or create mental images of a stimulus in a highly precise manner. Thereby, HP participants might be able to engage in deeper and more complex processing of the perceptual elements of the visual stimuli during encoding, which in turn improves their ability to retrieve these stimuli during the mental search processes involved in recognition.

With the observed correlations between brain activity during incidental encoding and behavioral performance during the recognition phase, we provide direct experimental evidence for the relationship between FFG activity during incidental encoding of facial stimuli and subsequent memory performance. These results are quite relevant for structure-function relationships because we could demonstrate that already during incidental encoding there are differences in content-specific brain regions, which directly correlate with behavioral performance differences during recognition. This indicates that sensory processing and content-specific brain regions might play a more important role for performance differences in memory tasks than previously thought, specifically when using incidental encoding tasks. Within the context of memory as a highly complex process with different sub-stages, our findings illustrate that during early stages (i.e., sensory processing), before explicit storage or retrieval processes have taken place, brain activity differences can predict later recognition performance.

## Conclusions and Future Directions

In the present study, SDT-derived measures of behavioral recognition performance correlated with brain activity during incidental encoding in regions that are specialized for detection of salient stimuli (insula), stimulus-driven attention (TPJ), and content-specific processing of mnemonic stimuli (FFG). We suggest that HP exhibited better memory performance due to both, increased content-specific activity in sensory processing areas, and more content-independent activity in higher order attention and salience detection areas. Our results provide first evidence that increased activity during incidental encoding in early visual areas is correlated to improved behavioral performance during recognition. This conclusion is particularly interesting given the basic processing features of some of these regions (e.g., FFG). We conclude from these findings that apart from storing mnemonic representations, sensory processing of stimuli to be encoded and the subsequent supply of attentional resources that help to highlight certain stimuli to be remembered is highly important for appropriate memory performance—even in an incidental setting.

To go beyond the correlative evidence presented here, future studies should aim at further clarifying the causal role of both content-specific sensory processing areas and content-independent attention and salience detection areas for memory performance. This could be achieved by using non-invasive brain stimulation to inhibit activity within one of these areas during incidental encoding. Furthermore, patients with lesions specifically affecting the insula, TPJ, or FFG should be compared to patients with lesions in the MTL region, to assess how different lesions affect memory performance. Finally, we would like to reiterate that the present results were obtained using an incidental encoding memory task without explicit instructions to remember stimuli. Interestingly, this indicates that brain activity differences in both content-specific sensory processing areas and content-independent attention and salience detection areas might constitute general differences in encoding ability and memory performance between HP and LP.

## Conflict of Interest Statement

The authors declare that the research was conducted in the absence of any commercial or financial relationships that could be construed as a potential conflict of interest.
